# A new forensic tool to date human blood pools

**DOI:** 10.1038/s41598-020-65465-4

**Published:** 2020-05-25

**Authors:** F. R. Smith, C. Nicloux, D. Brutin

**Affiliations:** 1 Aix-Marseille University, IUSTI UMR CNRS 7343, 13007 Marseille, France; 2 Institut de Recherche Criminelle de la Gendarmerie Nationale, 95300 Pontoise, France, Cergy, France; 3 Institut Universitaire de France, 75231 Paris, France

**Keywords:** Applied physics, Biological physics, Fluid dynamics

## Abstract

Courtrooms are asking for reliable scientific evidence in order to prevent wrongful convictions. Thus, a more rigorous approach to forensic science approved by scientific methods is promoted. The study of human blood dynamics in the context of forensic science is becoming a widespread research topic, although the physics behind wetting and drying of blood is not completely understood. Based on the morphological changes of drying blood pools, the following work presents a patentable method to quantitatively date these blood pools for forensic purposes. As for drying drops of blood, cracking patterns are observed but they are more disordered. Similar disordered crack patterns are observed in the case of gels, their evaporation process is, therefore, presented since this topic has been thoroughly investigated. We aim to find reliable patterns that could give information concerning the evolution of a blood pool over time to lead to practical application of this knowledge. An empirical model is established between final dried blood patterns and the generating mechanism, yielding application in bloodstain pattern analysis for forensic investigations.

## Introduction

Bloodstain pattern analysis is the forensic speciality dedicated to the analysis of blood traces found on a crime scene^1^. After a bloodshed event, a blood pool may be found on the scene. Although some first suppositions from the observation of blood pools can be deduced, the physics concerning the drying processes of blood pools is not yet well known. Some previous studies have focused on the volume determination of a blood pool for reconstruction purposes^2–5^ or to determine if such blood loss could be the cause of death^6^, but the temporal question of when a blood pool was formed remains unanswered. The current methods used on a crime scene to find the time at which events occurred are body temperature, rigor mortis, forensic entomology, etc., methods for which the presence of a body is necessary. If no body is present, the time estimation of a blood pool formation becomes a crucial piece of information. In other cases, it would complement the other available information. Presently, no such method exists. The aim of the present study is therefore to predict the time at which a blood pool is formed with an accuracy of ±30 mins in order to add a new piece of information to the global timeline. Furthermore, the evaporation dynamics of volumes of blood greater than a drop have not yet been investigated; therefore, understanding the evaporation mechanisms of blood pools introduces aspects of fluid mechanics of blood which could serve in practical application, bringing a more rigorous approach to forensic interpretation^7^.

**Figure 1:**
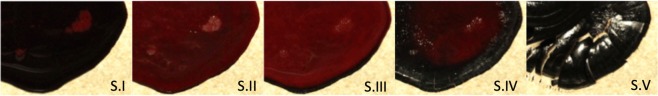
Time-lapse of a drying pool of blood from a healthy person, at 23 °C with a relative humidity of 20% showing the drying stages.

Prior work on the evaporation of blood pools examined the morphological changes of blood pools and distinguished five distinct stages^8^, as shown in Fig. 1:

- (S.I) coagulation stage
- (S.II) gelation stage
- (S.III) rim desiccation stage
- (S.IV) centre desiccation stage
- (S.V) final desiccation stage

It highlighted as well the fact that a pool does not dry in a uniform manner, supporting the idea that the shape of the pool is critical in the dynamics of evaporation. To monitor the evolution of drying pools, the study suggested following the drying front, corresponding to the transition edge between wet and dry blood, and visually refers to the transition between the red and the black colour. This drying front represents an interesting feature to follow with image processing. To understand the morphological observation of the drying front and to develop a reliable tool of interpretation, an understanding of the evaporation mechanism is, therefore, needed.

## Evaporation principles

Evaporation corresponds to a mass transfer process of liquid into vapour, which takes place at the liquid/vapour interface. This process can take place if the gas phase is not yet saturated, corresponding to a thermodynamic non-equilibrium. The evaporation of drops with a contact angle of 90° or less have been widely studied^9–12^ and it has been shown that the evaporation depends on the contact line dynamics and is proportional to the radius of the drop. However, for larger stretches of liquids such as a lake, the evaporation is no longer influenced by the triple line. The evaporation can be described as a steady state regime where the flux goes from region of high concentration towards region of lower concentration along a concentration gradient. This is therefore a one dimensional situation, which is described by Fick’s law:

1 $$J=-D\frac{d\varphi }{dx}$$

where J is the diffusion flux in *mol*.*m*^−2^.*s*^−1^ and corresponds to the amount of substance that flows through a square meter during a second, D is the diffusion coefficient in *m*^−2^.*s*^−1^, *φ* is the concentration in *mol*.*m*^−3^ and *x* is the length dimension expressed in *m*. In the case of blood pools, the evaporation appears to be in an intermediate situation between the evaporation of a drop, and one dimensional evaporation. The height of the pool decreases during evaporation which corresponds to the one dimensional dynamic, but then desiccation occurs first at the triple line, where the rim dries first. Then this drying front propagates towards the centre of the pool. Actually, to describe the shape of a liquid, the capillary length, *λ*_*c*_, is often referred to in fluid mechanics. In general, the order of magnitude of *λ*_*c*_ for most liquids is about a few millimetres. In the case of drops, the radius, which is the characteristic length of this shape, is of the order of *λ*_*c*_, indicating that surface tension prevails over gravity. In the case of larger stretch of liquid (ponds,…) the depth is this time the characteristic length of the shape and is much greater than *λ*_*c*_, indicating that gravity prevails. For the considered pools of this study, an intermediate model has therefore to be considered, since gravity forces flatten the surface in a pancake shape^13^, but the edges present a contact angle with the surface. Consequently, to tackle the evaporation problem of a pool, parameters such as the thickness of the pool or its shape have to be taken into account. Indeed different shapes will imply different evaporation mechanisms at the triple line. Additional parameters concerning the surroundings have to be considered as well during evaporation, such as the surface, the humidity, and the temperature. From a physical point of view, blood is generally described as a biphasic liquid, with water as the fluid matrix and red blood cells (RBCs) as spherical elastic particles of 8 *μ* m^14–17^. Therefore blood is comparable to a colloidal suspension where the interactions between the RBCs are dominated by short range interactions and gravitational forces are negligible. This is comparable to a sol where the interactions between the particles exhibit a Brownian motion. When blood is deposited on a surface, RBCs are evenly distributed and move freely in the aqueous phase. Then blood being *ex-vivo*, haemostasis occurs rapidly induced by the aggregation of platelets that form a temporary sealant while fibrinogen is being converted to a network of fibrin polymers. Coagulation is known as being the innate response to stem bleeding. It is this response and the formation of the fibrin web that lead to the gelation of the phase. Following gelation, drying takes place with evaporation first, then diffusion and finally shrinkage.

**Figure 2:**
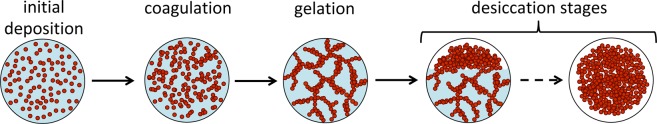
Overview of the different steps of blood evaporation. The filled red circles represent the RBCs while the blue represents the plasma.

These different stages are presented in the Fig. 2. These different steps of evaporation recall sol-gel processing with first the formation of a gel, evaporation from the surface followed by evaporation through the porous media. Finally cracking is observed as well. To understand the evaporation processes of blood, some analogy between a sol-gel transition and blood pools drying could, therefore, be envisaged.

## Sol-gel transition and drying

A sol is a colloidal suspension, where the dispersed phase corresponds to solid particles exhibiting Brownian motion. When there is a chemical reaction or during drying, it then evolves into a gel-like diphasic system containing a liquid phase and a solid phase. For the gel-like properties to appear, some of the liquid phase may have to be removed by evaporation, depending on the volume fraction of the particles present in the sol. Since the literature concerning blood pools drying is scarce, but many studies describe the drying of gels^18^, we reviewed the literature of sol-gel transitions in order to later compare it with drying blood pools and see if some similarities exist.

One of the first observations concerning the sol-gel transition is that the process goes through different stages. First the sol transforms into a gel, which presents viscous and elastic properties. The clusters grown either by polymeric condensation or by particle aggregation assemble together to form a giant cluster called gel. Once the gel has formed, evaporation then takes place through this porous media. First the body of the system shrinks and the volume loss corresponds to the volume that has evaporated. After a critical point the body becomes rigid and stops shrinking. The liquid then recedes inside leaving near the surface air-filled pores. However evaporation continues to occur at the surface. Finally when only a small amount of liquid is left in some isolated pores, evaporation can only take place inside the system followed by vapour diffusion to the outside. Consequently drying of gels can be divided into several drying stages. Already in 1986 Dwivedi examined this process in some detail by studying the drying process of alumina gels of different thicknesses focusing on the mass loss over time. As a result he found that about 7% of the initial mass of gel is left after drying. In the case of blood pools, it was similarly shown that 23% of the initial mass is left after drying^8^. Dwivedi then looked at the different drying stages and found that alumina gels undergo three drying stages. The first stage is a constant rate period (CRP)^19–21^ where the volume decrease of the gel is equal to the volume of liquid lost by evaporation. In this stage, the evaporation rate appears to be comparable to that of an open dish of water^22^. A critical point is reached at the end of this stage, inducing the shrinkage to stop. Subsequently to this, cracking of the gel is more likely to occur. After the critical point, gels undergo a first falling rate (FRP1) where the liquid flows through partially empty pores, followed by a second falling rate (FRP2) corresponding to the final stage of drying. In this final stage, evaporation occurs inside the body and the liquid diffuses to the surface in the form of vapour. The description of the sol-gel transition and the drying of inorganic gels represent a classic work where the mechanisms are well established^19^. Therefore, examining its characteristics is interesting since we can envisage that the drying process of blood is likely to present some similarities, which will now be presented.

## Materials and methods

### Blood properties

A certified nurse sampled blood in 9 ml dried evacuated collection tubes. Blood collection was carried out and approved in accordance with relevant guidelines and regulations of the university and of the National Scientific Research Council. Informed consents were obtained from all volunteers. The tubes are neutral tubes, without any anticoagulant or activator, and pools are created within 30 seconds after sampling in order to prevent anticoagulant agents interacting with the action of fibrinogen and clotting and to recreate real bleeding events. The blood is slowly deposited from the tube onto the surface, a few millimetres above the surface. An additional blood tube was sampled for heamatological analysis, but in a coated tube with an anticoagulant. Using heamatological analysis (Mindray, BC 3600), the heamatocrit value is determined. For women the heamatocrit level was between 36.2 ± 0.1% and 42.6 ± 0.1%, and for men between 40.1 ± 0.1% and 47.1 ± 0.1%.

### Experimental set-up

In order to accurately follow the drying of a blood pool, the environment must be controlled and kept as constant as possible. Thus, blood pools were created in a glovebox (Jacomex T-Box, V = 350 L) where the humidity can be controlled. The experimental room was regulated by air conditioning, allowing the temperature to remain constant. Three different surfaces were used, a linoleum surface, a varnished wooden flooring surface and a tile. The chosen surfaces are non-porous surfaces commonly found in crime scenes. The initial mass of the pools varied between 0.30 ± 0.01 g to 31.37 ± 0.01 g. To measure the initial masses, and the mass loss during drying, the surfaces were placed on a balance (Mettler Toledo, MS6002TS). For the first set of experiments only one balance and one surface were used, but for the second set, another surface was placed on another balance (Mettler Toledo, MS6002TS) next to the first one. The first set of experiments was to identify the different phases of the drying process. The aim of the second set was to compare two pools drying in the same conditions of either same weight but with different shapes or of same shape but different weights. A camera (EOS 7D digital camera, resolution: 5184 × 3456 pixels) was suspended above the pools and pictures were recorded every two minutes. A surface reference length was placed next to the pools in order to determine later their surface areas.

## Results and discussion

### Thermodynamic aspects of drying

**Figure 3:**
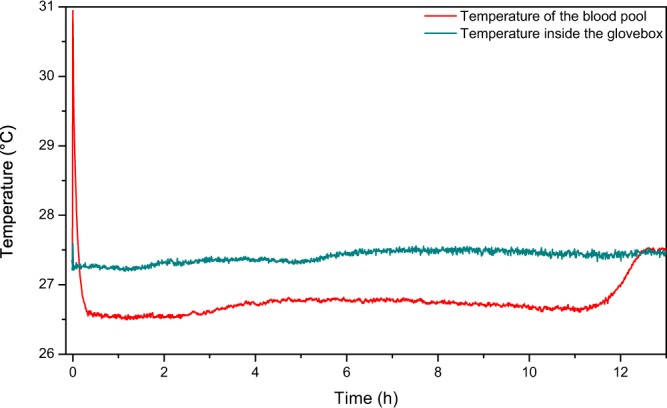
Recorded temperature of a drying blood pool inside the glovebox and of the temperature just above this same pool; *m*_*i*_ = 8.75 g, HCT = 46.7, and 20% humidity.

Drying implies two types of transfer: heat transfer from the surroundings towards the liquid and a mass transfer from the system towards its surface and then into the surrounding air. Blood temperature inside the body varies from 36 °C to 37.5 °C. To observe the temperature changes of blood when taken from the body, two thermocouple probes were placed, one in the blood pool, at the centre of the pool, and another one just above the blood pool. The temperature in the glove box was kept constant at 27.2 °C. Once blood was deposited on the surface, there is first a very fast increase of the temperature measured by the probe. It reaches 31 °C. This corresponds to the transient heat transfer of the warm blood touching the surface at room temperature. The temperature decreases then rapidly and reaches a plateau of 26.4 °C. It takes about 25 mins for the blood to reach this plateau value, which could be attributed to the heat diffusion/convection inside the pool. Then the observed plateau appears to be approximatively 1 °C lower than the temperature measured just above the pool itself as shown in Fig. 3. This is in agreement with blood evaporation as an endothermic process. This temperature difference is stable over time, until the pool starts cracking. Once the cracks reach the probe placed in the pool, the probe is no longer in the liquid part of blood, and the measured temperature goes back up to the ambient temperature. This temperature is the same one as the one measured with the other thermocouple placed just above the pool.

### Evaporation dynamics

**Figure 4:**
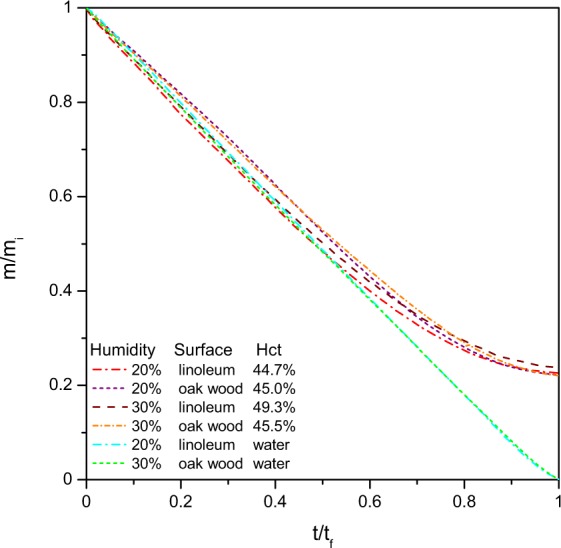
Normalised mass as a function of normalised time of the blood and water pools. Experiments were carried out at 20 or 30% humidity, on linoleum or oak wood surfaces. The heamatocrit values of the blood ranged between 44.7 and 49.3%.

Blood is comparable to a colloidal solution where plasma corresponds to the aqueous phase since plasma is a water based solution, composed of 90 to 92% water, of 7 to 8% plasma proteins and the last 1 to 2% are trace amounts of other constituents^23^. Therefore a first simple study was made to follow the mass loss of the blood pools over time and to compare them to pools of water. Natural water evaporation is of course a well known phenomenon, but to our best knowledge actual research papers have focused mainly on natural evaporation from open water, ponds or water baths but no work has focused on the drying of a water pool formed by natural spreading. To compare water evaporation to blood evaporation, blood and water pools were created in parallel and dried in exactly the same conditions. Two different surfaces, linoleum and varnished oak wood, were tested in two different humidities of 20% and 30%. Since the pools had different drying durations, *t*_*f*_, and initial masses, *m*_*i*_, the obtained results were normalised as presented in Fig. 4 in order to compare both types of pools.

The initial mass loss for water or blood pools is linear until it reaches approximatively 60% of mass loss. At this stage, the pool is pinned to the surface and the liquid phase evaporates at the surface. The volume loss corresponds then to a decrease in height. When for water the mass loss keeps diminishing in a linear manner until 0% of mass is left, for blood pools the mass flow rate decreases significantly until the mass left corresponds to approximatively 23% of the initial pool mass. This amount corresponds to the biological deposit, explaining the small variations that are observed according to the initial heamtocrit value. The same percentage was already observed in the case of blood drops^24^.

**Figure 5:**
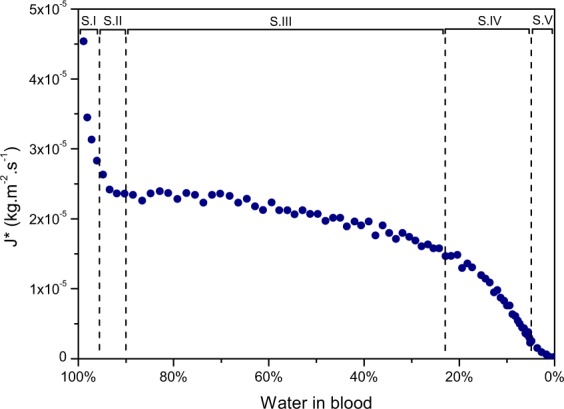
Rate of water loss against the water contained in the pool for a blood pool; *m*_*i*_ = 31.37 g, HCT = 42.1%, T = 23.9 °C and 20% humidity.

To investigate further the mass loss over time of the pools, evaporation rates were experimentally analysed. To observe an evaporation rate, the mass loss of a pool, which corresponds to a water mass loss, is evaluated over time per unit area. The pools were weighed at 6 to 12 min intervals during the initial drying stages and 20 to 30 min intervals at later drying stages. The evaporation rate corresponds to the recorded mass loss over the time interval per unit area of the total pool area. The evaporation rate is named J* since it corresponds to a mass diffusion flux. Figure 5 presents the obtained water evaporation rate of a blood pool of initial mass *m*_*i*_ = 31.37 g as a function of the percentage of water left in the blood. The pool was dried at a temperature of 23.9 °C and at a humidity of 20%.

By looking at the evaporation rate of the blood pool in Fig. 5 as a function of water left, the evaporation can be compared to the dynamics of alumina gels, where the evaporation rate as a function of percentage of water left was examined^22^. Some similarities are clearly noticeable. Just after the pool formation, the rate of water loss decreases fast until coagulation of the phase S.I and then reaches a constant rate until the end of phase S.III. This recalls the first drying phase of gels, CRP. Then there are successively two rate decreases as well, S.IV and S.V, similar to the falling rate periods FRP1 and FRP2, until all the water present has fully evaporated. During these last stages, evaporation no longer takes place at the surface but within the porous media. The original liquid is then transported in the form of vapour to the outside, which explains the decrease in the evaporation rate. In the final stage, cracks start being observed. The crack patterns of an entire blood pool are quite different from a single blood drop, where the cracks are elongated and directed towards the centre of the stain. Nonetheless these cracks show similarities to the ones observed by Pauchard *et al*.^25^ during the drying of a colloidal suspension having an ionic force of I = 0.4 mol.L^−1^ or the drying of latex particles 0.1 *μ*m in suspension^26^. These cracks follow the drying front and the separation between them depends on the thickness of the sample, the physical and chemical properties, the adhesion with the surface and on the desiccation conditions^26,27^. Although the main mechanisms and evaporation of a blood pool seem to be similar to that of a gel, the characteristics of a pool have to be analysed as well since in practical cases the shape and size could vary significantly.

#### Shape influence on the evaporation rate

In order to study the influence of the shape of a pool on its drying, Twenty pools of the same initial mass, 5.24 ± 0.47 g, but of different shapes were created. These were dried in the same humidity of 20%, at the same temperature of 23 ± 1 °C and on the same surface, a white tile. The initial mass, *m*_*i*_ of a pool is known, thus the volume, *V*, can be calculated using the blood density:

2 $$V=\frac{{m}_{i}}{\rho }$$

The area, *A*, and the perimeter, *P*, of each pool were measured using the imageJ software. From this, the height, *h*, of the pool can be estimated with the following approximation:

3 $$h=\frac{V}{A}$$

**Figure 6:**
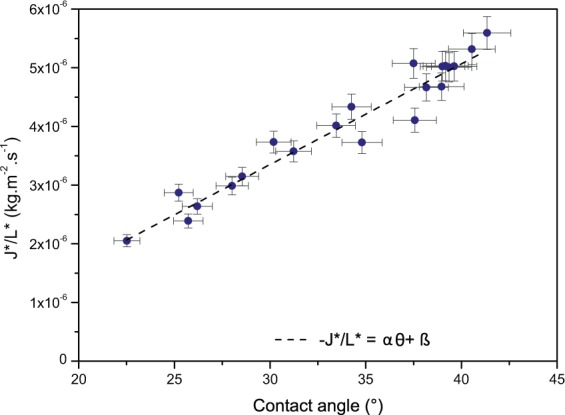
Evaporation rates for blood pools of initial masses 5.24 ± 0.47 g divided by their corresponding shape factors L* as a function of the contact angle of the pools. The pools were dried at 20% humidity, T = 23 ± 1 °C, on the same surface, a tile. The dashed line corresponds to the linear relationship.

These dimensions characterise a blood pool. An easy dimensionless factor can therefore be deduced, which correlates these shape properties. This shape factor named L* is simply defined according to the following equation:

4 $${L}^{\ast }=\frac{A}{hP}$$

with *A*, the area, *h*, the height and *P*, the perimeter of the pool. The shape factor L* was calculated for the twenty pools. An average evaporation rate for each pool was also estimated after 50% of the initial mass had evaporated. This was in order not to have results biased by the final stage of evaporation, which is very slow and could induce some errors in the overall rate estimation. The obtained evaporation rates were different for each pool despite the fact that the pools had a similar volume, and were drying in similar conditions. To take into account the shape factor, the evaporation rates (in *kg*.*m*^−2^.*s*^−1^) of the pools were then divided by the shape factor L*. As a result a linear variation of the modified evaporation rates as a function of the contact angle of the pool was observed (Fig. 6).

The linear variation is described by: J*/L* = *α*   *θ* + *β*, with *α* = 1.72.10^−7^ and *β* = 1.79.10^−6^. To calculate the contact angle of the pool the following approximation^8^ was used:

5 $$\theta =\arccos \left(\frac{g{m}_{i}^{2}}{2\gamma \rho {A}_{i}^{2}}+1\right)$$

The obtained results in Fig. 6 support the idea that the shape of the pool has an influence on the drying dynamics and can be expressed in the form of a linear relationship. This linear relationship is valid for pools of similar weight, drying at same humidity and temperature.

#### Size influence on the evaporation rate

**Figure 7:**
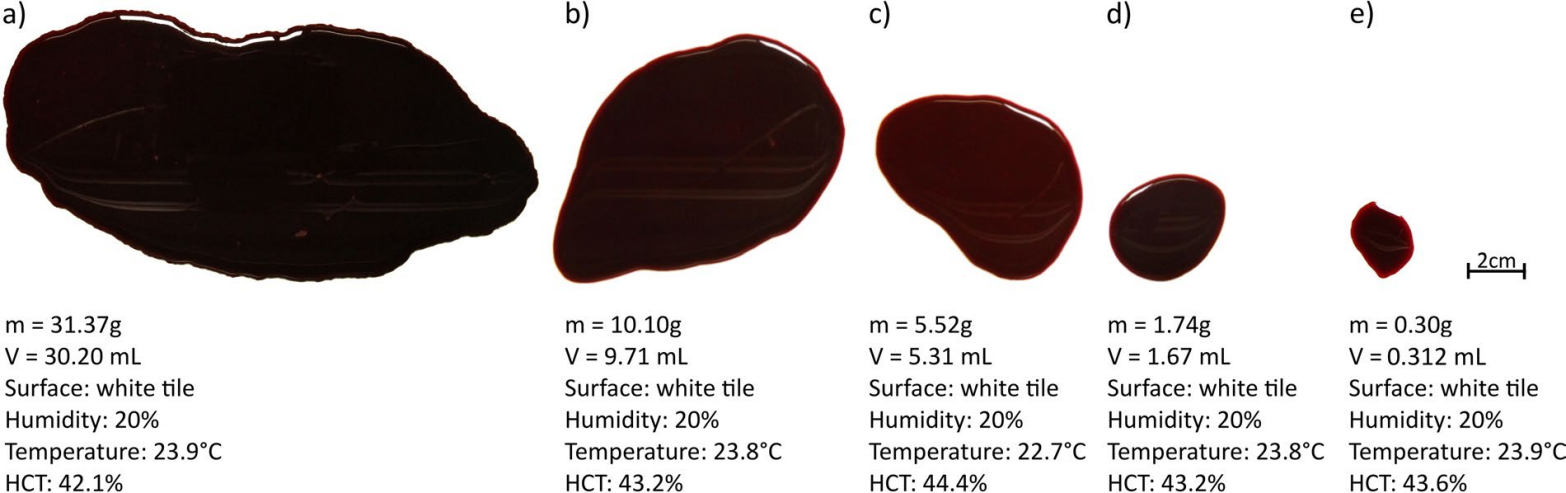
Characteristics of the five different pools used to illustrate the study on the influence of the size.

To study the influence of the size of a pool on its evaporation rate, we compared pools of different initial masses drying in similar conditions, i.e. at the same humidity of 20% and the similar temperatures of 23 ± 1 °C on the same surface, a white tile. In Fig. 7, five pools have been selected, whose characteristics are given.

**Figure 8:**
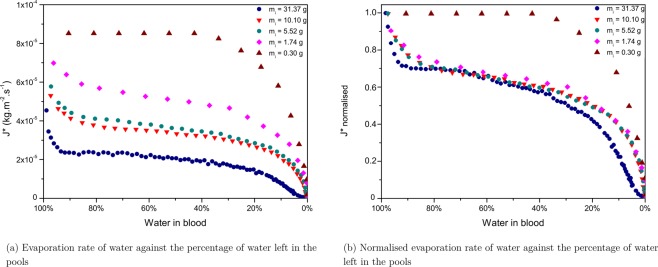
(**a**) Evaporation rates and (**b**) Normalised evaporation rates of pools having various initial masses (*m*_*i*_), drying at 20% humidity, T = 23 ± 1 °C, on the same surface, a tile.

The rate of water loss as a function of water left in blood for the five pools is compared in Fig. 8a, and in a normalised way in Fig. 8b. The normalised results show obvious different dynamics for the pools having a very low *m*_*i*_ compare to the other pools exhibiting very similar evaporation dynamics. The smallest pool of 0.30 g approaches the size of a large drop. It presents the highest evaporation rate. Moreover the dynamics present first a plateau evaporation rate until a sharp decrease in the evaporation rate is observed at the very end. The evaporation dynamics of deposited blood drops are said to be dependent on the contact angle, the vapour concentration and the radius of the drop. The total drying time of a deposited drop of blood being relatively short (of the order of half an hour) the observed final pattern is the results of a time-scale competition between the thermo-capillary convection and the movement of the particles that takes places in evaporating drops of blood. Some studies have emphasised the role played by Marangoni flow to keep the evaporation flux constant in the first evaporation stage^28^. After deposition, RBCs are homogeneously dispersed inside the drop. The radial flow triggers the redistribution of RBCs towards the periphery of drops leading to the biological deposit^24^. After a critical evaporation point and clotting (occurring from 2 to 8 min after deposition), Marangoni flow is no longer possible inside the drop and RBCs are trapped into the central area of the drop. As a result, the evaporation rates decreases significantly until complete evaporation of the drop. This model appears to no longer be valid for larger amounts of blood, as can be seen on Fig. 8b where the evaporation stages exhibit different dynamics for the other pools. The evaporation rate of water loss appears to decrease as the mass of the pools increases (Fig. 8a). For smaller blood volumes, evaporation is faster. This could be explained by the humidity directly surrounding the pool coming from the change of state of the aqueous phase into vapour and which is less significant for small blood volumes. Inversely, the greater the size of the pool is, the more difficult it becomes for the aqueous phase to change into vapour since the surface is already saturated with vapour. However, Fig. 8b shows that all pools undergo the same evaporation stages, from the small pool of *m*_*i*_ of 1.74 g to the large pool of *m*_*i*_ 31.37 g.

The area considered for the evaporation rate corresponds to the total area of the pool, but when the pool starts desiccating the wet area decreases, which explains the slow decrease of the evaporation rates towards the end of evaporation, when about 25% of water is left in blood. This resembles the dynamics observed in the case of gels suggesting that with increasing blood volume, there is a smooth transition between drop-like evaporation dynamics to gel-like evaporation dynamics. The fact that the final falling rate is mainly visible for the largest pool strengthens the assumption that pools of greater volumes tend towards gel-like evaporation dynamics. This graph illustrates very well how increasing the volume of a pool affects its evaporation rate.

#### Overcoming size and shape influences

**Figure 9:**
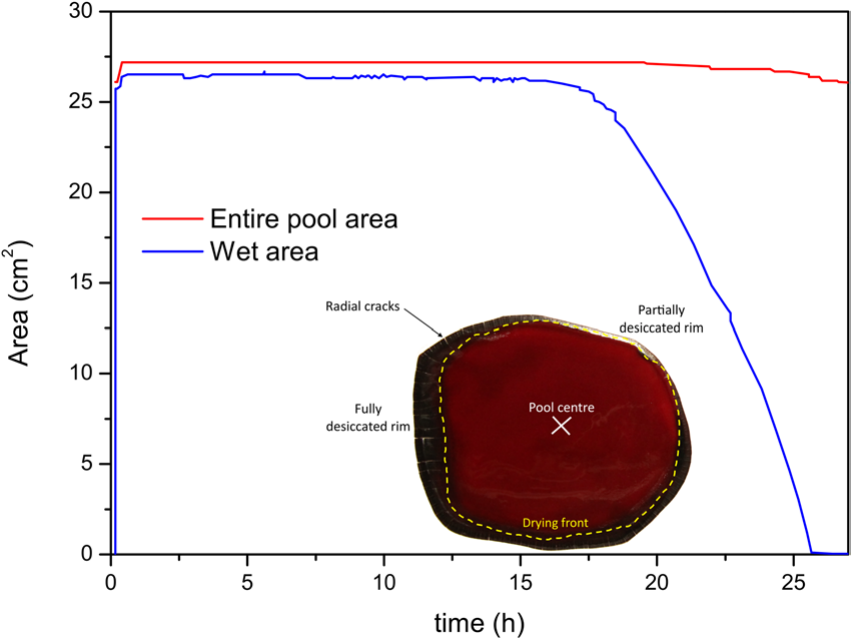
In red: area as a function of time of the entire pool; in blue: wet area of the pool as a function of time; *m*_*i*_ = 5.41 g, HCT = 40.0%, T = 22 °C and 60% humidity.

In the previous section the evaporation rate of water was calculated using the flow rate per unit area, using the total area of the pool. To overcome the influence of the size of the pool, it could be envisaged to calculate the evaporation using this time the wet area. By using the photographic analysis that were recorded during the experiment, the area of the entire pool and of the wet area of the pool could be measured as a function of time as illustrated with one pool in Fig. 9. In this figure the entire area of the pool remains constant with time except after 25 h corresponding to shrinkage of the pool when cracking occurs. The wet area, delimitated by the drying front, is constant at the beginning and then decreases in an almost linear manner.

**Figure 10:**
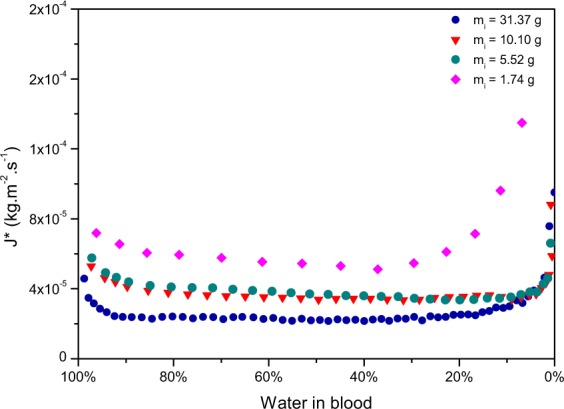
Evaporation rate of water against the percentage of water left in pools of various initial masses (*m*_*i*_), drying at 20% humidity, T = 23 ± 1 °C, on the same surface, a tile.

To calculate the evaporation rates using the wet areas instead of the total areas, the measurements of the wet areas of the four pools (a), (b), (c) and (d) were used. Pool (e), was excluded for this part of the study since its evaporation dynamics were closer to the one observed for drops. The obtained evaporation rates are presented in Fig. 10. The evaporation rates of water appear now to be constant. Although, at the very beginning and at the end of the process, the values no longer follow this plateau. Before blood coagulation, the evaporation dynamics are likely to approach the dynamics of water, whereas after coagulation those dynamics should be closer to the dynamics of a gel, which explains that there’s a small decrease at the beginning until reaching a plateau. Then, the evaluation of the evaporation rates is biased towards the end since the image analysis software can not yet determine very precisely the wet area when it comes to the final stage of drying.

This constant value of the evaporation rate of water as a function of water left in blood implies that using the area analysis the dynamics can be predicted. Nonetheless the plateau appears to be different for the four pools. Several parameters must act. A simple relation describing the evaporation rate of a free surface based on convection already exists, given by^29^:

6 $${J}^{\ast }={K}_{i}\frac{MPw}{RT}$$

with *K*_*i*_ the transfer coefficient of the liquid state into the gaseous state (in *m*.*s*^−1^), *M*, the molar mass (in *kg*.*mol*^−1^), *P*_*w*_, the saturation vapour pressure of water at the surface (in *J*.*m*^−3^), *R* the universal gas constant (in *J*.*mol*^−1^.K^−1^), and *T*, the temperature (in K). This equation shows that in the case of evaporation of a free surface, the evaporation rate is dependent on the saturation vapour pressure and on the humidity. This equation can be rewritten as:

7 $${J}^{\ast }\frac{RT}{M{P}_{w}}={K}_{i}$$

**Figure 11:**
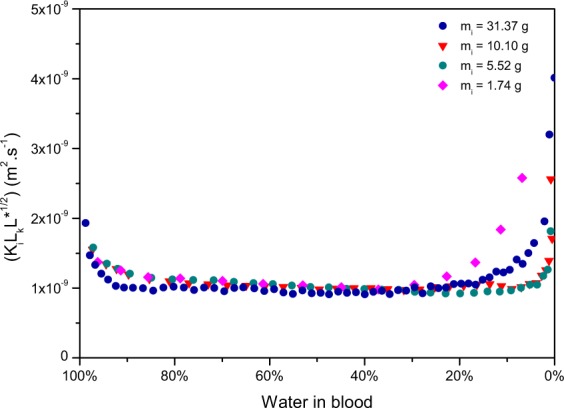
Evaporation rate of water weighted by RTL_*k*_ L*^1/2^/M *P*_*w*_ against the percentage of water left in pools having diffirent initial masses (*m*_*i*_), drying at 20% humidity, T = 23 ± 1 °C, on the same surface, a tile.

The obtained value of the transfer coefficient *K*_*i*_ is in *m*.*s*^−1^. In order to approach the evaporation of blood pools with Fick’s Law, the diffusion must be described. Therefore a diffusion coefficient (in *m*^2^.*s*^−1^) should be analysed instead of a transfer coefficient (in *m*.*s*^−1^). So, to obtain a diffusion coefficient of the pools, the transfer coefficient was multiplied by a characteristic length. The Knudsen layer, *L*_*K*_, was used as characteristic length since it corresponds to the very thin evaporation layer of vapour near the liquid, which is described by the following equation:

8 $${L}_{k}=\frac{kT}{\pi {d}^{2}{P}_{a}}$$

where *k* is Boltzmann’s constant, *T*, is the temperature, *d* is the molecular diameter and *P*_*a*_ is the atmospheric pressure. Therefore to obtain the diffusion coefficient of each pool, *K*_*i*_ should be multiplied by the corresponding *L*_*K*_. In the case of blood pools, evaporation depends on the shape of the pool, since the evaporation rate is modified compared to an infinite stretch of fluid. To compare the calculated diffusion coefficient of the pools, the shape factor should be taken into account. As a results the evaporation rate is weighted by the universal gas constant *R*, the temperature *T*, the molar mass, *M*, the saturation vapour pressure, *P*_*w*_, the Knudsen layer, *L*_*K*_, and in fact by the square root of the shape factor L*. As a result, the plateaus observed in Fig. 10 scale together to a unique plateau of approximately constant value of 1.10^−9^ *m*^2^.*s*^−1^ as shown in Fig. 11.

This plateau could bring an approximation of the diffusion coefficient of blood pools. It appears to be lower than the one measured for a drop of blood^30^ of 9.8.10^−8^ *m*^2^.*s*^−1^ but in good agreement with the values obtained for diffusion of colloidal gel of stacked particles into air^31^, where values ranged between 10^−9^ and 10^−10^ *m*^2^.*s*^−1^.

The various characteristics of a pool added to the variability of the surrounding parameters lead thus to a very complex problem. It appears that all these parameters are related together, as would suggest the results presented in Fig. 11. This last result presents a very interesting approximation suggesting that drying blood pools are very similar to gels. Although the pools were dried in similar conditions, mainly same humidity and same temperature, these parameters should be considered in further studies, as they are known to have an influence on evaporation. With temperature increase, diffusion coefficients are known to increase. This suggests that temperature can affect greatly the evaporation rates. Relative humidity affects evaporation as well since diffusion coefficients are known to decrease as the relative humidity increases. Further research should be devoted to seek out on how could temperature affect the plateau obtained for pools drying at 23 ± 1 °C. Fundamentally it can be accepted that the shape of a pool and the surroundings parameters are key to understand the evaporation rates. Undeniably the geometry affects the evaporation rate since some geometry would favour evaporation. For two pools of identical volume, the round pool will have its Knudsen layer saturated in vapour faster than the thin and stretched pool. The evaporation of the thin pool will be favoured, it will thus have a greater evaporation rate. Although estimating the geometry of a pool exhibits some uncertainty, the fact of taking the geometry in consideration already erases a large amount of errors. Moreover the obtained correlation can find very interesting practical application.

### Forensic interpretation

**Figure 12:**
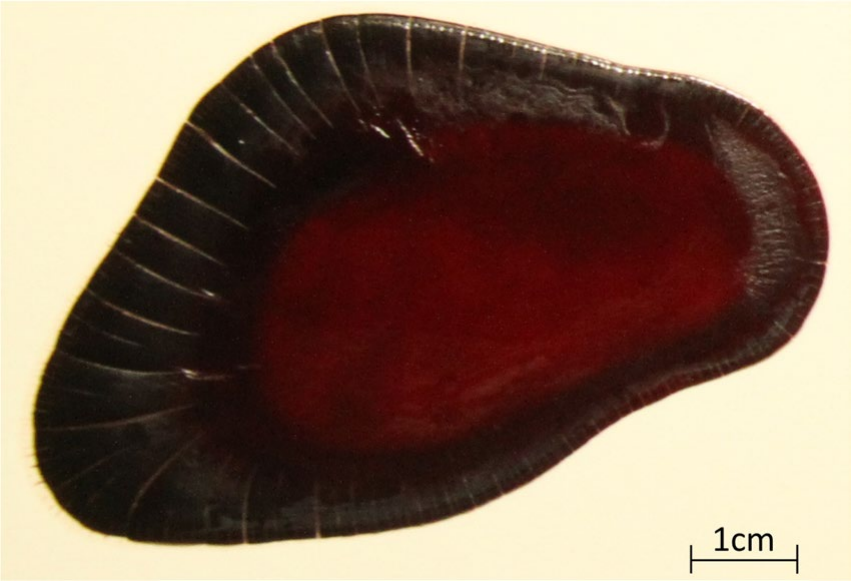
Pool drying at 22.5 °C on a white tile.

The observations made during this study highlight the complexity of the problem of drying blood pools. Although many questions remain, some interesting features have been characterised, especially the drying front. This particular feature presents an interest to BPA analysts since it can easily be monitored by photography. In practice, investigation teams need tools, which can easily be brought on the scene, and that will not induce an alteration of the evidence. Photography is a tool being already used. The aim of this work was thus to develop a method that would require only photographies so that it could be concretely applicable. Using this practical field tool, and the previously obtained constant value of the diffusion coefficient for drying blood pools, the time at which a pool was formed can be calculated for a pool drying in the same conditions. The pool presented in Fig. 12 was dried at 22.5 °C on a white tile. The drying front is already clearly visible in this photograph.

First, the evaporation rate J^*^ of this pool can be calculated using the approximate constant value D_*blood*_ = 1.10^−9^ *m*^2^.*s*^−1^, corresponding to the plateau observed in Fig. 11.

9 $${J}^{\ast }={D}_{blood}\frac{M{P}_{w}}{{L}_{k}RT{L}^{\ast \mathrm{1/2}}}$$

L^*^ is obtained with

10 $${L}^{\ast }={A}_{i}/hP$$

where *A*_*i*_ is the total area of the pool, and P its perimeter. Both can be measured in the picture of Fig. 12. *h* is estimated from the average value of the height measured for this surface for 30 different pools and yields a value of 1.44 ± 0.19 mm. Next, to correlate the calculated evaporation rate with the elapsed time, *δ*t, since this pool was formed, the mass variation is needed. *m*_*i*_ can be obtained with

11 $${m}_{i}=h{A}_{i}\rho $$

**Figure 13:**
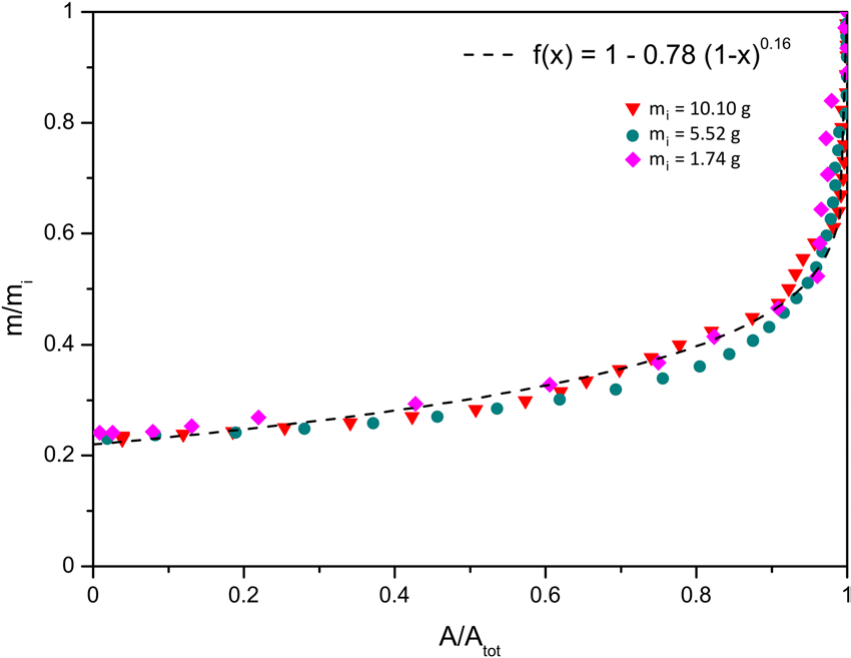
Normalised mass as a function of normalised wet area for different pools having different initial masses (*m*_*i*_), drying at 20% humidity, T = 23 ± 1 °C, on the same surface, a tile.

Then *t*_*x*_ corresponds to the time at which the picture was taken after pool formation such that *δ* t = *t*_*x*_ − *t*_*i*_. *m*_*x*_ is the mass of the pool at *t*_*x*_. Since the wet area, *A*_*x*_, at *t*_*x*_ can be measured, a relation between area and mass is necessary. The following graph in Fig. 13 presents the normalised area as a function of the normalised mass for the pools studied in the previous section.

Again, when normalised, to a first approximation, the curves overlap each other, corresponding to the fitting function: *m*/*m*_*i*_ = 1 − *α*[1 − (*A*/*A*_*i*_)]^*β*^, with *α* = 0.78 and *β* = 0.16 This function gives an approximative relation between the wet area and the mass of the pool. Hence, from *A*_*x*_, *m*_*x*_ is obtained. Finally *t*_*x*_ can be estimated using

12 $$\delta t=\frac{\delta m}{{J}^{\ast }{A}_{i}}$$

with *δ*m = *m*_*i*_ − *m*_*x*_. Given *t*_*i*_ = 0 s, the expression of *t*_*x*_ is:

13 $${t}_{x}=\frac{\alpha R{k}_{B}{T}^{2}{A}_{i}^{\mathrm{1/2}}{h}^{\mathrm{1/2}}\rho {\mathrm{[1}-({A}_{x}/{A}_{i})]}^{\beta }}{M{d}^{2}\pi {P}^{\mathrm{1/2}}{D}_{blood}{P}_{w}{P}_{a}}$$

A drying time of 8 h 18 min was calculated for the photograph in Fig. 12, which was only 19 min less than the exact time 8 h 37 min. The process was repeated for another pool dried in the same conditions, and a difference of 5% was obtained.

By repeating this process several times as the pool dries, an average value would reduce the uncertainty. This first result of calculation of the time at which a pool was formed is a promising result, suggesting that it will become possible to calculate the time at which a pool was formed on a crime scene. For now, many precautions should be taken. This calculation worked for a controlled environment. Anyhow, by further investigating the different variables, mainly the temperature which can influence the evaporation rate, some reference values could be established to create a reference table. A set up has already been envisaged that would only require a camera. Pictures of the drying front of the pool in different areas, depending on the accessibility, could be automatically taken at a regular interval (such as every 5 or 10 min). This could be done for a couple of hours and then after image processing, a statistically valid value could be obtained with a margin of error. Therefore, if a reference table existed for the different temperatures, humidity, and surfaces, a defined protocol could be set up. Although, work should be done in order to evaluate if analysing only some parts of the advancing front gives equivalent results as for entire blood pools. The method presents as well some limitations, since to work, the drying front must be present. This means, that if the pool is already fully dried, the method can no longer be applied. On the contrary, if the pool is still entirely wet, then the investigation teams would have to wait for the drying front to appear. Thus, further work has now to be focused on the influence of the previously described parameters, and on the development of a very precise image processing tool. Indeed, since the difference between wet area and dried area is dependent on the image analysis, future development should seek to improve this tool.

## Conclusions and perspectives

In this study the evaporation dynamics of blood have been described in detail and some similarities with the sol-gel transition were found. Indeed blood is similar to a colloidal suspension with RBCs being the dispersed phase, and plasma the aqueous phase. After coagulation and fibrin precipitation, blood forms a gel like system that will then dry following different evaporation rates. This evaporation rate has been compared to the evaporation rate of pools of water drying in similar conditions. Finally, some of the pool characteristics were assessed such as the size and the shape of the pool. A blood pool is an intermediate system between a drop and a larger stretch of liquid. The latter corresponds to a system involving one dimensional heat and mass transfers. For the blood pool the evaporation at the triple line influences the entire evaporation rate. As a result we found that the shape influences the rate, which is expressed in a shape factor. The size of a pool, of course interferes with evaporation since, the greater is the volume that has to evaporate. Moreover, the drying front of the pool evolves in a non-uniform manner since it can start evolving towards the centre on one side while on the other side the pool is still in a gel like phase. Ultimately, all these parameters are linked to the advancing drying front. We have investigated the evolution of the drying front and related it to the evaporation rate of water, J^*^. By using the wet area of the pool to calculate J^*^, an almost constant value of the evaporation rate was observed. Then by incorporating the different variables that act int the evaporation process of a pool, an approximative constant value of diffusion coefficient was observed for pools of various sizes and shapes, but drying in the same conditions. Using this approximation, it became possible to calculate the time at which a pool, dried in the same conditions, was formed. Nonetheless, this last correlation should be investigated further for more pools, of different shapes and larger sizes, and drying at different temperatures and humidities. Some effort on the research should be turned towards this issue as it could then bring a modelling of the evaporation of blood pools, which could then be monitored on a crime scene simply by following the drying front and using a reference table. This study used blood from healthy volunteers, males and females with haematocrit values ranging from 36.2 to 47.1%. Individual biological parameters did not show any significant influence on the evaporation dynamics. However other physiological parameters such illnesses, like haemophilia, or the intake of drugs, like aspirin, could have an indirect influence on evaporation since they are known to interfere with clotting. Future work on individual physiological aspects would thus improve the proposed model. Nonetheless, this investigation has demonstrated pioneering results concerning the drying of blood pools from healthy subjects, which may find exploitation in forensic analysis.

The following work is going through a patent application at the European Patent Office. The inventors of this patent are F. Smith, C. Nicloux, D. Brutin. The application number is EP19305772.6. The patent was filed on the 17th of June 2019. The patent application covers the forensic interpretation section.
